# Repeated and multiple fecal microbiota transplantations plus partial enteral nutrition as the first-line treatment in active pediatric Crohn’s disease

**DOI:** 10.3389/fcimb.2023.1083236

**Published:** 2023-02-23

**Authors:** Biao Zou, Shengxuan Liu, Xuesong Li, Jiayi He, Chen Dong, Mengling Ruan, Zhihua Huang, Sainan Shu

**Affiliations:** Pediatric Department, Tongji Hospital, Tongji Medical College, Huazhong University of Science and Technology, Wuhan, Hubei, China

**Keywords:** fecal microbiota transplantation, partial enteral nutrition, Crohn’s disease, first-line treatment, pediatric

## Abstract

**Background:**

Most studies have reported fecal microbiota transplantation (FMT) as an effective secondary option for Crohn’s disease (CD). However, there is little data on FMT as a first-line treatment for CD. In our study we explore the rates of clinical and endoscopic remission and mucosal healing after FMT plus partial enteral nutrition (PEN), as a first-line treatment for active CD in children.

**Methods:**

We retrospectively enrolled pediatric CD patients who underwent PEN or PEN plus FMT treatment at diagnosis from November 2016 to July 2019 at the Pediatric Department, Tongji Hospital. The two groups were defined as FMT group (repeated and multiple doses of FMT plus PEN) or PEN group (PEN alone). All the patients received PEN intervention. At baseline and week 8- 10, the FMT group was administered multiple doses of FMT to help induce and maintain remission. All patients were evaluated at week 8- 10 and 18-22 *via* clinical and relevant laboratory parameters and endoscopic results. The clinical and endoscopic remission and mucosal healing rates were compared between the two groups at different time points after the therapy.

**Results:**

Twenty-five newly diagnosed active CD patients were included in the study, containing 7 females and 18 males with a median age of 11. 1 ± 2.3 years. 13 and 12 patients were assigned to the PEN and FMT groups, respectively. At week 8-10, clinical remission was obtained in 83.3% and 53.8% of the FMT and PEN groups, respectively (p=0.202). The endoscopic remission rates were 72.7% for FMT and 25.0% for PEN (p=0.039), whereas the mucosal healing rates were 27.2% for FMT and 0% for PEN (p=0.093). At week 18-22, clinical remission was achieved in 72.7% and 20.0% of patients in the FMT and PEN groups, respectively (p=0.03). Theendoscopic remission rates were 66.6% and 12.5% in the FMT and PEN groups, respectively (p=0.05), whereas the mucosal healing rates were 55.5% and 0% in FMT and PEN groups, respectively (p=0.029).

**Conclusion:**

This study demonstrate that FMT plus PEN can be used as a first-line treatment for active CD in children.

## Introduction

Crohn’s disease (CD) is an acute and chronic nonspecific inflammatory gastrointestinal disorder. In recent years, with the westernization of diet and lifestyle, as well as the widespread use of antibacterial agents and heavy social pressure, CD has become increasingly common worldwide; in China, and the incidence is becoming increasingly in younger (Wang et al., 2013; Ng et al., 2017). Compared to adults, the course of CD in children is more rapid, the disease changes faster, and the intestinal tract is more extensively involved, which seriously affects growth and development (Kuenzig et al., 2022).

Currently, the main treatment methods for CD include exclusive enteral nutrition (EEN), oral corticosteroids, immunity inhibitors, and biologicals. Corticosteroids are critical in the induction of active CD remission, but are unable to sustain long-term treatment with serious adverse reactions. Immunity inhibitors, such as methotrexate and azathioprine, which are used for CD maintenance in children, have significant side effects. Biological agents, such as infliximab and adalimumab are relatively effective in treating CD in children. However, long-term potential adverse events, such as infection and high cost, greatly limit their widespread use. Multiple studies have confirmed that EEN has a significant effect on inducing remission in mild-to-moderate pediatric CD (Buchanan et al., 2009; Frivolt et al., 2014; Moriczi et al., 2020), but foods should be strictly avoided making EEN unsuitable as an effective means of maintaining remission. Partial enteral nutrition (PEN) is a more patient-friendly and tolerant treatment (Johnson et al., 2006), and PEN alone is generally considered partially effective, but PEN in combination with medication has been shown to be beneficial in inducing and maintaining remission (Takagi et al., 2006). Although its etiology is not fully understood, gut imbalance and dysregulation of immunological responses plays a critical role in the development of CD (Gevers et al., 2014). As the best way to regulate intestinal flora, fecal microbiota transplantation (FMT) is recommended as a guideline for therapy of recurrent Clostridium difficile infections (Cammarota et al., 2017).

Published evidences have demonstrated that FMT is one of the effective ways to treat CD (Goyal et al., 2018; Green et al., 2020; Sokol et al., 2020). Most of these studies have addressed FMT as an alternative for the first-line treatment for CD. Data have shown that the therapeutic efficacy of FMT declines over time, and the timing of the second FMT helps maintain the long-term benefit of active CD (Cui et al., 2021). To date, no studies have evaluated FMT coupled with PEN as a first-line treatment in inducing and sustaining remission in CD with children. We conducted a retrospective study to evaluate the clinical remission and mucosal healing *via* repeated and multiple doses of FMT plus PEN (80%), as the first-line treatment in pediatric patients with CD. The study served as a pilot study to NCT05321758 which was registered as a clinical trial.

## Methods

### Ethics

The present study was approved by the Medical Ethics Committee of Tongji Hospital, Tongji Medical College, Huazhong University of Science and Technology (TJ-C20220313). Written informed consent for FMT treatment was obtained from parents or legal guardians of all pediatric subjects.

### Study design

We retrospectively reviewed the medical charts of children with mild-to-moderate active CD who were hospitalized in the Department of Pediatrics in Tongji Hospital of Tongji Medical College, Huazhong University of Science and Technology from November 2016 to July 2019. The diagnosis of CD was rested on history, clinical symptoms, endoscopy, and histological evidence.

The inclusion criteria met the following: (i) age of 6–14 years with no genetic diseases; (ii) all newly diagnosed with mild-to-moderate CD with early PEN (80%) and/or FMT treatment; (iii) in addition to the PEN and FMT treatment, other treatments should not be added. Exclusion criteria included: (i) children who were treated with PEN (80%) for less than eight weeks or with other drugs during that time, including corticosteroids, methotrexate, thiopurines, and anti-TNF agents; and (ii) incomplete data for patients.

**Figure 1:**
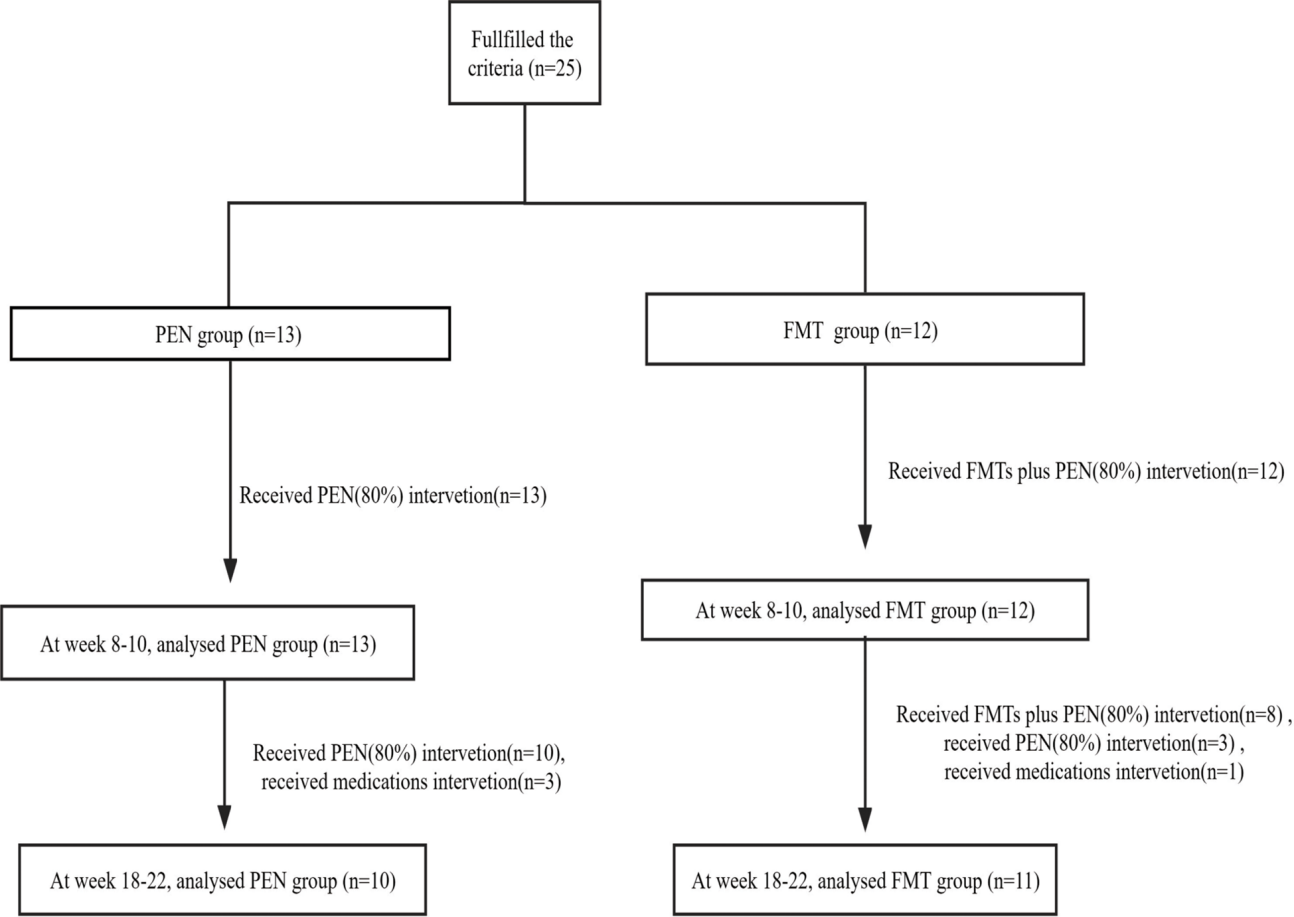
Flowchart of patient inclusion. FMT, fecal microbiota transplantation; PEN, Partial enteral nutrition.

All children with CD received PEN (80% of total calories as a pooled diet, Peptamen, Nestle, Vevey, and swiss) intervention at diagnosis to help induce and maintain clinical remission, and the FMT group received multiple FMT interventions at baseline and week 8- 10 in addition to PEN treatment (**Figure 1**). The volume of formula per day was calculated according to the estimated energy requirement (EER) × 120% (basal rate plus an additional 20% caloric needs for weight gain). The EER was calculated based on the recommendations of the Chinese Dietary Reference Intake. Another 20% of the calories comes from a regular diet with no allergens and limited animal protein.

Patients treated with FMT coupled with PEN were defined as the FMT group, and those treated with PEN alone served as the PEN group. Patients with mild-to-moderate CD, defined by the Pediatric Crohn’s Disease Activity Index (PCDAI) of >10 and ≤40, and Simple Endoscopic Score for CD (SES-CD) of >3, were enrolled in the study. The Paris classification was used to assess the anatomical location and behavior of the disease (Levine et al., 2011). Clinical remission was defined as a PCDAI score of ≤10 (Grover et al., 2016). Endoscopic remission was defined as SES-CD ≤2 and PCDAI score ≤ 10 (Vuitton et al., 2016). Mucosal healing was defined as SES-CD=0 and PCDAI score ≤ 10 (Peyrin-Biroulet et al., 2015).

The time point at which CD was diagnosed was the baseline. Colonoscopy was conducted by trained pediatric endoscopists at baseline and at two assessment points (week 8- 10 and 18-22) after the therapy, and SES-CD scores were calculated jointly by two pediatric attending physicians. If the participants required additional medication within 18 weeks, they were considered clinically invalid and were not included in the next evaluation point.

### Donor screening

12 healthy donors were recruited in the study with one recipient and one donor. Eligible donors, including children with similar age (e.g., relatives and trusted friends), were recruited according to the following criteria (Kelly et al., 2015; Cammarota et al., 2017; Liu et al., 2017): (i) no history of infectious conditions (e.g., tuberculosis); (ii) no history of metabolic syndromes (e.g., diabetes); (iii) no gastrointestinal illnesses and functional disorders, including chronic fatigue and irritable bowel syndrome (IBS); (iv) no allergic diseases (e.g., eczema); (v) no antibiotic taken in the past three months; (vi) no autoimmune diseases; (vii) no drug abuse history; and (viii) on a regular diet during donation of the material.

To minimize the risk of disease transmission, the donors underwent rigorous serological and stool tests within 10 days before the FMT donation. Clinical tests, including complete blood count, biochemistry, hepatitis virus, HIV, and common intestinal pathogens, should be normal to qualify as donors (**Supplementary Table 1**). They had to rescreen and re-evaluate any abnormalities in the symptoms and signs. Fecal 16S RNA or macrogene sequencing was performed if necessary.

### FMT procedure

**Figure 2:**
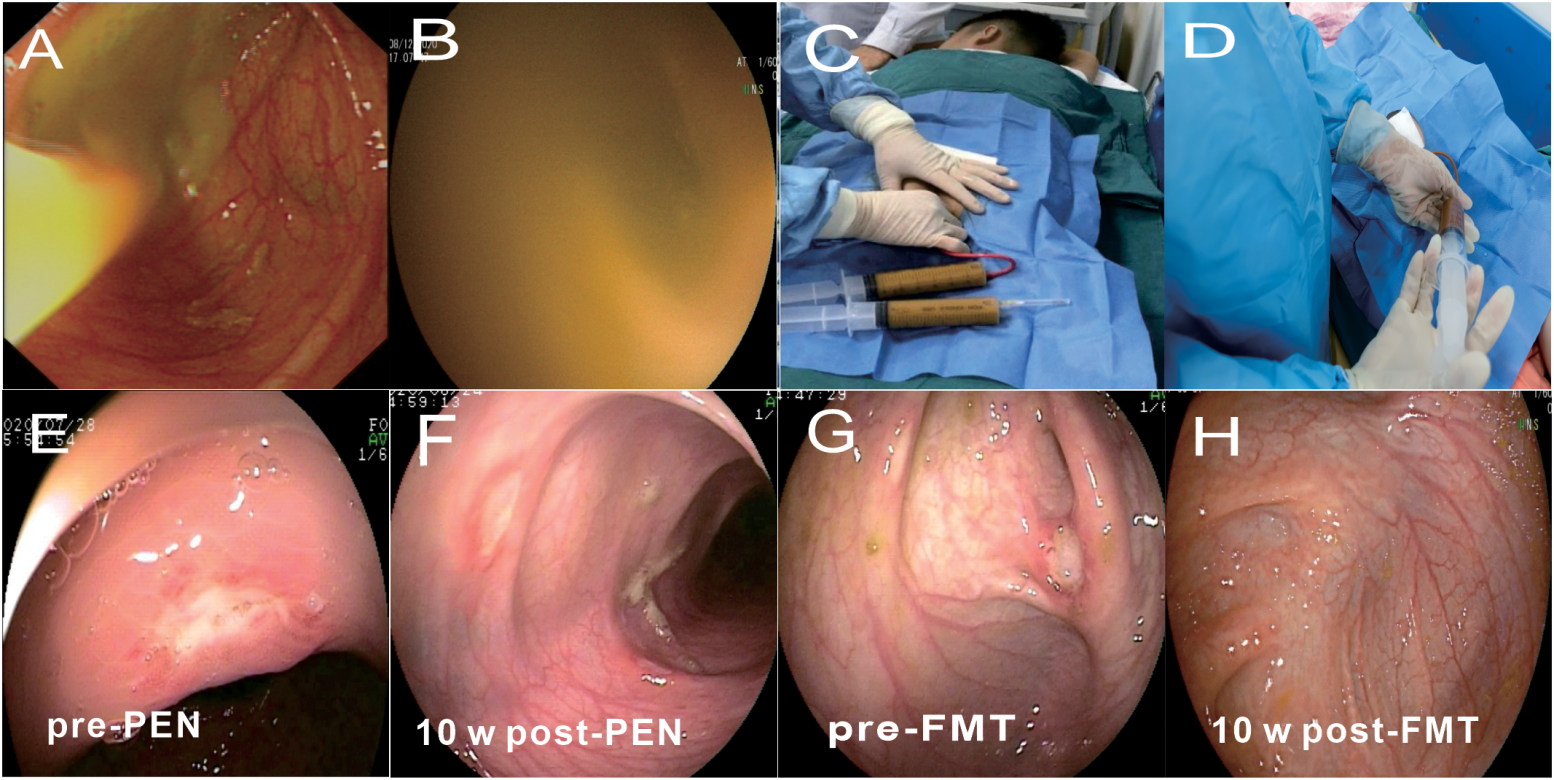
Delivery route of fecal microbiota transplantation (FMT) and related clinical response. **(A)** Endoscopic images show microbiota suspension being infused. **(B)** Endoscopic images showed no visible particles when the colonoscope was inserted into the infused microbiota suspension at the end of the ileum. c,d Fecal microbiota transplantation (FMT) were performed by retention enema (**C**, Retention enema, **D**, Infused microbiota suspension under the retention enema). **(E, F)**The change of endoscopic appearance pre-PEN and 10 weeks post-PEN. **(G, H)** The change of endoscopic appearance pre-FMT and 10 weeks post-FMT.

12 patients received therapy with FMT plus PEN (80%). The guardians of the 12 patients refused immunological interventions, considering the adverse effects of corticosteroids and immunosuppressants, and they agreed to FMT as first-line therapy. The number of FMT infusions was grouped into single (1 day) or multiple infusions (2-10 days continuously). No bowel preparation (cleanup or laxative administration) was performed before the FMT. The donor feces were collected 1 h pre-FMT, attenuated, and mingled with sterile normal saline (1 mg of feces was attenuated with 5ml of saline). Samples were filtered through sterile gauze, and a 100mL fresh fecal microbiota suspension was prepared for the FMT. The fecal suspension was poured into a sterile cup for the FMT procedure within 1 h. The routes of administration included colonoscopy and enema. Fecal microbiota transplantation (FMT) were performed by colonoscopy (**Figures 2A, B**). Fecal microbiota transplantation (FMT) were performed by retention enema (**Figures 2C, D**). After infusion, the patients were asked to hold a fixed position (>25° semi-reclining or hip-up position) for at least 4 h. The FMT procedure followed a uniform standard for each patient. All the patients in the FMT group received fresh fecal suspensions.

### Microbiota analysis

Fecal microbiota was analyzed for two patients before and after FMT. DNA extracted from feces collected from the healthy donors, and patients pre-FMT (one day before FMT), at week 8 and week 18 of after FMT using a PowerSoil DNA Isolation Kit (Mo Bio Laboratories, Carlsbad, CA). 16S rRNA gene (V3-V4 region) were amplified using PCR primers as described previously (Liu et al., 2017). Diversity and changes of fecal microbiota were analysed on the QIIME2 v.2020.2 platform.

### Data collection

The data of twenty-five pediatric patients used in this study were extracted from medical records. To aid in evaluating clinical efficacy and safety, laboratory data were collected at baseline (at week 0), at week 8–10 and 18–22, including laboratory inflammation and biochemical indicators, such as erythrocyte sedimentation rate (ESR), C-reactive protein (CRP), hemoglobin (Hb), leukocyte, serum albumin, platelet, vitamin D, fecal calprotectin, PCDAI, SES-CD score, and FMT-related adverse events (AEs).

### Statistical analysis

Continuous variables are presented as mean (standard deviation) or median (range). Noncontinuous parameters are depicted as frequencies and percentages. According to the results acquired, nonparametric test (Mann Whitney U test) or a parametric test (T test) were used to evaluate differences between groups. Univariate analysis between the two groups was conducted using the Chi-square test or Fisher exact test for categorical data. The IBM SPSS Statistics 25 was used for the statistical analyses. Statistical significance was set at p<0.05 in a two -side test. GraphPad Prism version 8 was used to draw graphics.

## Results

### Patient characteristics

From November 2016 to July 2019, twenty-five pediatric patients were analysed in this study, containing seven females and eighteen males with a median age of 11. 1 ± 2.3 years. All patients enrolled in the current study were newly diagnosed CD and treated with PEN (80%) for more than eight weeks. Of those, 13 patients belonged to the PEN and 12 in the FMT group, respectively. There were no statistically remarkable differences in the baseline demographics between the two groups (**Table 1**).

**Table 1:** The baseline characteristics of the study population at the time of diagnosis.

Parameters	FMT group	PEN group	P value
Total number	12	13	-
Age(year), M± SD (range)	10.69 ± 2.39	11.56 ± 2. 16	0.319
Sex, female, n (%)	3(0.25)	4(0.31)	1
Disease duration before PEN(mo)	0.068 ± 0.009	0.074 ± 0.015	0.325
Hemoglobin (g/L)	109.6 ± 21.9	107.7± 13.5	0.868
White cell count (109/L)	11.7± 1.6	13.9± 1.5	0.336
Platelet (109/L)	416 ± 43	383 ± 25	0.525
Vitamin D	17.4 ± 4.0	16.7 ± 5.5	0.525
ESR	31.5 ± 22.4	33.5± 18. 1	0.813
CRP (mg/L)	33.9 ± 34.7	33.5 ± 25.3	0.644
Albumin (g/L)	35.9 ± 5.3	35.4 ± 4.9	0.781
Disease location			0.595
L1 Terminal ileum	5 (41.7%)	6 (46.2)	
L2 Colon	3 (0.25%)	6 (46.2)	
L3 Ileocolonic	4 (0.33%)	2 (15.3%)	
+L4 (upper GI tract)	2 (16.7%)	1 (7.7%)	
Disease behaviour			1
B1 non-stricturing or non-penetrating	10 (83.3%)	10 (76.9%)	
B2 stricturing	2 (16.7%)	3 (23. 1%)	
B3 penetrating	0	0	
Perianal involvement	2 (16.7%)	2 (15.3%)	1
PCDAI score	23.2 ± 6.7	23.5 ± 5.6	0.909
SES-CD score	6.75± 1.05	6.54± 1.05	0.62

CRP, C-reactive protein; ESR,erythrocyte sedimentation rate; PCDAI, Pediatric CD Activity Index; SES-CD, Simple Endoscopic Score for CD.

The median time from CD diagnosis (at baseline) to PEN initiation (80%) was 0.07 ± 0.01 months. Twenty-two patients (88%) received PEN orally at all time, but three (12%) patients required nasogastric feeding for the first five days. The guardians of the FMT group all consented to FMT as their first-line treatment. The median time from CD diagnosis to FMT initiation was 0.31 ± 0.09 months in the FMT group. The data of the children in the FMT group are shown in **Table 2**.

**Table 2:** Factors related to FMT in patients treated with FMT.

Patient	CDI	EBV	CMV	Median time from CD diagnosis to FMT initiation (mo)	Times of FMT (baseline)	Times of FMT (week 8- 10)	Activity Index (PCDAI )			SES-CD			Fecal calprotectin ( µg/g)			Patient-donor relationship	Donor age(yr)
							baseline	week 8 – 10	Week 18 –22	baseline	week 8 – 10	Week 18 –22	baseline	week 8 – 10	Week 18 –22		
P1	–	–	–	0.5	1	/	22	30	/	7	9	/	/	/	/	relative	9
P2	–	–	–	0.2	8	7	20	2	0	6	2	2	/	/	/	friends	9
P3	–	–	–	0.3	1	/	30	5	15	8	2	0	/	/	/	friends	15
P4	–	–	–	0.5	9	10	37	5	5	9	2	0	3330	480	128	relative	12.7
P5	–	–	–	0.2	3	2	32	5	5	7	0	0	1890	166	108	relative	9
P6	–	–	–	0.3	10	/	20	15	25	7	2	7	1093	160	966	friends	13
P7	–	–	–	0.3	9	7	17	0	27	7	0	8	1980		736	relative	10
P8	–	–	–	0.3	7	7	27	5	10	7	3	3	1880	120	710	relative	14
P9	–	–	–	0.3	10	7	20	5	2	6	2	0	/	/	/	friends	12
P10	–	–	–	0.3	4	/	22	5	5	6	0	/	/	/	/	relative	9
P11	–	–	–	0.3	9	4	17	10	10	6	4	/	/	/	/	relative	12
P12	–	–	–	0.3	9	9	15	5	0	5	/	0	/	/	/	relative	7

FMT, fecal microbiota transplantation; CDI, C. difficile infection; EBV, EB virus; CMV, cytomegalovirus; PCDAI, Pediatric CD Activity Index.

Patients who received multiple doses of FMT usually once a day consecutively, underwent the first colonoscopy, and the others through an enema. In the PEN group, 13 patients were treated with PEN (80%) alone for more than 8 weeks, 10 of them were treated with PEN (80%) alone for more than 18 weeks, and another 3 patients were switched to biologics at weeks 8-10. In the FMT group, 12 patients received one round of FMT plus PEN (80%) therapy for more than 8 weeks, 8 of them received two rounds of FMT plus PEN (80%) therapy for more than 18 weeks, 3 of them received one round of FMT plus PEN (80%) therapy for more than 18 weeks, and another 1 patient switched to biologics at weeks 8- 10 (**Figure 1**). Of the 25 patients, only three had a PEN (80%) treatment duration longer than 24 weeks.

### Evaluation of induction remission (at week 8-10)

One patient in each group did not undergo endoscopic examination at week 8- 10 after the treatment. The induced clinical and endoscopic remissions and mucosal healing rates in the FMT group were remarkably higher than those in the PEN group (**Table 3**).

**Table 3:** Comparison of clinical and endoscopic outcomes between the two groups at different time points.

Times	Outcome	FMT group	PEN group	P value
8- 10w	Clinical remission (PCDAI ≤ 10) Endoscopic remission (SES-CD ≤ 2) Mucosal healing (MH) (SES-CD = 0) Clinical remission (PCDAI ≤ 10)	10/12(83.3%) 8/11(72.7%) 3/11(27.2%) 8/11(72.7%)	7/13(53.8%) 3/12(25.0%) 0 2/10(20.0%)	0.202 0.039 0.093 0.03
18-22w	Endoscopic remission (SES-CD ≤ 2) Mucosal healing (MH) (SES-CD = 0)	6/9(66.6%) 5/9(55.5%)	1/8(12.5%) 0	0.05 0.029

FMT, fecal microbiota transplantation; PEN, Partial enteral nutrition; PCDAI, Pediatric CD Activity Index; SES-CD, Simple Endoscopic Score for CD.

Clinical remission was observed in 10 (83.3%) and 7 (53.8%) patients in the FMT and PEN groups, respectively (p=0.202). The mean PCDAI score was decreased from 23.2 ± 6.7 at baseline to 7.6 ± 7. 1 in the FMT group, and from 23.5 ± 5.6 at baseline to 16.7 ± 11. 1 in the PEN group. Endoscopic remission was observed in 8 of 11 of the patients (72.7%) in the FMT group and 3 (25.0%) in the PEN group. **Figures 2E-H** shows the endoscopic appearance before and after treatment in FMT group and PEN group. Ulcers were still visible in the PEN group, but disappeared in the FMT group after treatment. This showed a better endoscopic remission rate in the FMT group.

Mucosal healing was observed in 3 of 11 patients (27.2%) in the FMT group and 0 (0%) in the PEN group. The mean SES-CD score was reduced from 6.75 ± 1.05 at baseline to 2.36 ± 1.9 in the FMT group, and from 6.54 ± 1.05 at baseline to 4.83 ± 2.8 in the PEN group.

**Figure 3:**
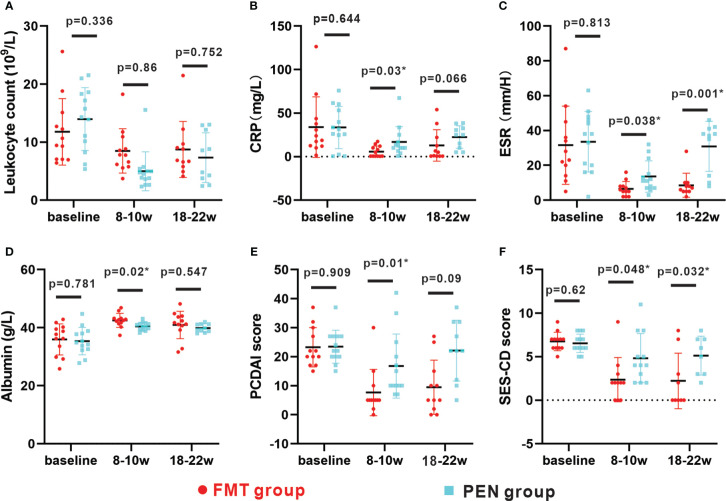
Changes in parameters between baseline, 8-10w and 18-22w in two groups. **(A–F)** Changes in leukocyte count, albumin, CRP, ESR, PCDAI score and SES-CD score between baseline, 8- 10w and 18-22w in the FMT group and PEN group respectively. The distribution of values within each group at each timing is illustrated by mean and SD. FMT, fecal microbiota transplantation; PEN, Partial enteral nutrition; CRP, C-reactive protein; ESR,erythrocyte sedimentation rate; PCDAI, Pediatric CD Activity IndexSES-CD, Simple Endoscopic Score for CD.

At week 8-10, there were significant statistical differences in PCDAI and SES-CD scores in both groups (both P<.05) (**Figure 3**). CD-related parameters such as ESR, CRP and serum albumin were evaluated, and both groups showed improvement from baseline. Compared with PEN group, FMT group showed more significant improvement, faster decline of ESR and CRP, faster increase of albumin, and better clinical efficacy. There were remarkable differences between the two groups in ESR, CRP and serum albumin (all P<.05) (**Figure 3**).

### Evaluation of maintaining remission (at week 18-22)

Three patients in the PEN group and one patient in the FMT group were switched to biologics after disease flare, leaving 11 patients in the FMT group and 10 patients in the PEN group to be analyzed at week 18-22. To help maintain remission, eight patients received multiple FMT doses again at week 8- 10 in the FMT group (**Table 2**).

The remission rate was remarkably better in the FMT group than in the PEN group (**Table 3**). In the FMT group, eight (72.7%) patients maintained clinical remission and seven (87.5%) of those received a second round of FMT treatment (**Table 2**). Only two (20.0%) patients maintained clinical remission in the PEN group. The mean PCDAI score was 9.45 ± 10.3 in the FMT group and 22. 1 ± 10.6 in the PEN group (p=0.09). The mean SES-CD score was 2.2 ± 1.06 in the FMT group, and 5. 1 ± 0.8 in the PEN group (p=0.032) (**Figure 3**). Endoscopic remission was observed in 6 of 9 (66.6%) patients in the FMT group and in 1 of 8 (12.5%) patients in the PEN group. Mucosal healing was observed in 5 of 9 (55.5%) patients in the FMT group and 0 (0%) patients in the PEN group. Two patients in each group did not undergo an endoscopic examination.

The mean values of ESR, CRP, PCDAI and SES-CD score in the PEN group were rebound between week 8- 10 and week 18-22. The mean PCDAI score was increased from 16.7 ± 11. 1 to 22. 1 ± 10.6, and the SES-CD score was increased from 4.83 ± 2.8 to 5. 1 ± 0.8, ESR and CRP also showed similar changes. However, These parameters of FMT group were relatively stable. There were also significant differences in ESR and SES-CD score between the two groups at week 18–22 (both P<.05) (**Figure 3**).These findings suggest that the PEN group is not effective in maintaining sustained remission and that FMT can help maintain clinical remission.

Fecal calprotectin (a cutoff <200 µg/g) of 5 patients in FMT group was significantly elevated, ranging between 1903 to 3330µg/g at baseline. Fecal calprotectin decreased significantly for between week 0 and week 8- 10. Fecal calprotectin of 2 children continued to decline between week 8- 10 and week 18-22, while fecal calprotectin increased in the other 2 children, which was considered to be related to disease flare (**Table 2**).

### Safety

A total of 21 FMT-related AEs occurred in 7 patients. Most AEs (90.5%) occurred within two days post-FMT. The AEs included abdominal pain(n=6), abdominal distension (n=5), nausea or vomiting (n=3), fever(n=2), constipation(n=2), diarrhea(n=2) and purpura (n= 1) (**Table 4**). One patient presented with abdominal pain and purpura in both lower limbs 12 hours after FMT. The diagnosis was allergic purpura. Abdominal pain improved one day after corticosteroid administration, which was considered to be caused by an immune disorder induced by bacterial flora. One patient developed fever with elevated blood routine and CRP levels 28 hour after the FMT. Inflammatory markers and body temperature returned to normal two days after antibiotic administration. The other AEs were self-limiting and symptom-free within 48 h.

**Table 4:** The adverse events of FMT.

Pt	Related AEs	Time from FMT	Causality	SAEs	Clinical treatment and outcome	Cured
1	abdominal pain abdominal distension nausea or vomiting	8h 8h 6h	Probable Probable Probable	× × ×	Self-improvement Self-improvement Self-improvement	√ √ √
2	abdominal pain purpura	6h 15h 6h 12h	Probable Probable Probable P b bl	× √ ×	Self-improvement Improvement after corticosteroid therapy Self-improvement Improvement after antibiotic	√ √ √
	abdominal pain diarrhea	8h 4h	ro a e Probable Probable	× ×	therapy Self-improvement Self-improvement	√ √ √
4	abdominal pain constipation abdominal distension	6h 52h 6h	Probable Probable Probable	× × ×	Self-improvement Self-improvement Self-improvement	√ √ √
5	abdominal pain nausea or vomiting fever	6h 3h 15h	Probable Probable Probable	× × ×	Self-improvement Self-improvement Self-improvement	√ √ √
6	abdominal pain diarrhea abdominal distension nausea or vomiting	8h 4h 8h 2h	Probable Probable Probable Probable	× × × ×	Self-improvement Self-improvement Self-improvement Self-improvement	√ √ √ √
7	Constipation abdominal distension	56h 6h	Probable Probable	× ×	Self-improvement Self-improvement	√ √

AEs, adverse events; SAEs, serious adverse events; FMT, fecal microbiota transplantation; h, hour.

### Microbial composition changes

**Figure 4:**
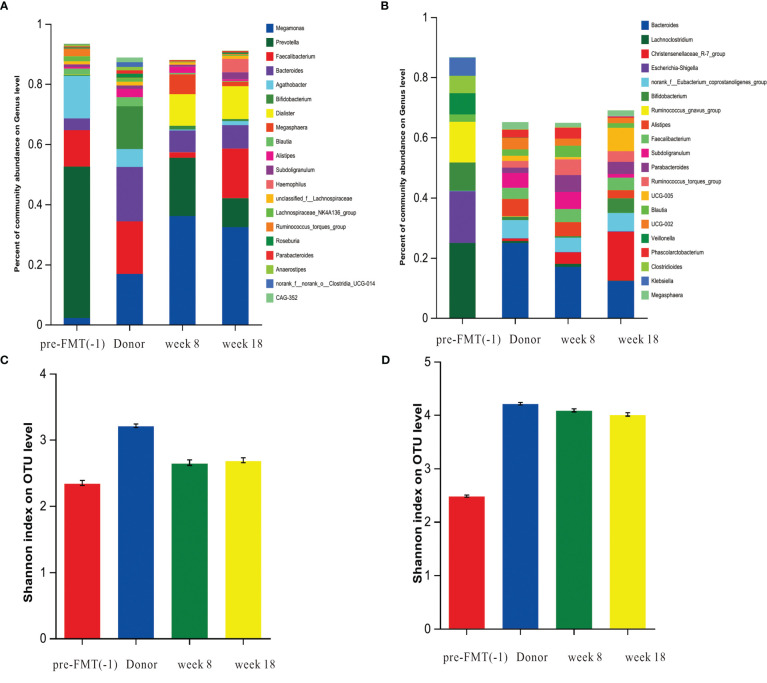
The dynamic changes in gut microbiota before and after FMT. DNA extracted from feces collected from the healthy donor, and recipient pre-FMT (-1), week 8, week 18 of after FMT was subjected to 16S rRNA gene (V3-V4 region) libraries construction and sequencing. **(A)** Species composition of feces samples of patient 5 at at genus level. **(B)**, Species composition of feces samples of patient 2 at genus level. **(C)** The Alpha diversity of patient 5 was measured by Shannon indexes. **(D)** The Alpha diversity of patient 2 was measured by Shannon indexes.

16s rDNA sequencing analysis showed that species richness and diversity of fecal microbial in 2 patients with Crohn’s disease were significantly lower than those of healthy donors. FMT treatment can increase the species richness and diversity of patients. The bacterial population of the recipient was close to that of the donor after FMT treatment (**Figure 4**). After FMT treatment, the relative abundance of bacterial genera *Bacteroides*, *Eubacterium*, *Parasutterella*, *Butyricicoccus* were significantly increased and *Clostridioides*, *Ruminococcus*, *Blautia* were decreased in 2 children (**Supplement 2**).

## Discussion

To the best of our knowledge, this is the first study to assess the effects of endoscopic remission and mucosal healing with repeated and multiple doses of FMT plus PEN as the first-line treatment in pediatric CD. The study indicated that within a small sample size of pediatric CD patients, repeated and multiple FMTs plus PEN can not only help induce and maintain remission but also contribute to mucosal healing.

Over the past decade, the therapeutic endpoint of CD has shifted from clinical remission to mucosal healing (Peyrin-Biroulet et al., 2015). The benefits of mucosal healing have been supported by clinical evidence to improve the long-term outcomes of CD patients, including reduced hormone use and incidence of risks, such as relapse and hospitalization (Ruemmele et al., 2015; Taylor et al., 2020). Previous FMT-related studies mainly focused on clinical remission (Goyal et al., 2018; Sokol et al., 2020; Cui et al., 2021), but paid little attention to mucosal healing. Our study showed that the mucosal healing rate in the FMT group were significantly higher than those in the PEN group in both the induction and maintenance stages, indicating that FMT treatment can promote mucosal healing. This is a very novel clinical result, which may be related to the ability of FMT can remodel gut microbiota and restore microbial diversity. Compared to that of donors, the gut microbiota diversity of pediatric CD patients was significantly decreased, and the abundances of *Eubacterium*, *Clostridia_ UCG-014*, *Oscillibacter*, *Coprostanoligenes* were lower, while the abundance of *Clostridioides* was higher in CD patients. After FMT, the species richness and diversity of our two patients showed significant improvement. FMT changed the relative abundances of several bacterial genera in 2 patients, including the relative abundance of *Bacteroides*, *Eubacterium*, *Parasutterella*, *Butyricicoccus* were significantly increased and *Clostridioides*, *Ruminococcus*, *Blautia* were decreased. We found that after FMT, the microbial community of children with CD was close to that of donors. This is why the endoscopic remission rate decreased from 72% to 66% but the mucosal healing rate increased from 27.2% to 55% at week 18-22.

Partial enteral nutrition uses the same liquid formulation as EEN, which significantly improves compliance and taste compared to EEN. Studies have suggested that PEN is beneficial in inducing and maintaining remission in patients with CD and more patient- friendly and tolerant treatment (Verburgt et al., 2021). Our study also confirms this view, but the effective duration is relatively short. In our study, ESR, CRP, SES-CD and PCDAI scores improved significantly between baseline and weeks 8- 10 in both groups, but FMT improved more. Between weeks 8- 10 and 18-22, these parameters remained largely unchanged in the FMT group and increased in the PEN group, after FMT treatment, fecal calprotectin also decreased significantly. All of this suggests that the FMT group was more successful in controlling inflammation. This indicates that the PEN treatment alone is poor in maintaining remission in CD patient, whereas FMT plus PEN is effective.

From the available evidence, to date, there is deficient evidence to recommend FMT as the first-line treatment for active pediatric CD patients. Knowledge on the application of FMT in pediatric CD are limited. Most of these studies have reported FMT as a secondary treatment option for refractory CD (Hourigan et al., 2015; Suskind et al., 2015; Goyal et al., 2018; Karolewska-Bochenek et al., 2018). Alka Goyal et al. (2018) reported that after a single FMT treatment, 57% of 21 children IBD patients who were refractory to conventional treatment showed clinical responses at 1 month. In a study by Suskind et al. (2015), 9 adolescents with CD were treated with a single FMT after refractory to conventional treatment, and 5 achieved clinical remission at 6 and 12 weeks. Karolewska-Bochenek K et al (Karolewska-Bochenek et al., 2018). reported that a two-week course of FMT was clinically effective in children with IBD refractory to standard therapy. Our study is the first paper to explore the first-line treatment of CD in children with FMT. Our data suggest that FMT combined with PEN therapy not only helps to induce remission in children with active CD, but also maintains clinical remission. This finding demonstrates that FMT may be a potential first-line treatment regimen for active pediatric CD. Given the drawbacks of current CD treatments, this should be a meaningful practice and exploration.

Several studies have demonstrated that a single FMT is safe and effective in patients with CD; meanwhile, multiple doses of FMT have been shown to be more effective than a single dose (He et al., 2017; Baunwall et al., 2020). Our findings are consistent with these results. Among the 12 patients in the FMT group, only two received a single FMT, and one (50%) was in clinical remission at week 8- 10. The other 10 patients received multiple FMTs, of which nine (90%) showed clinical remission at at week 8- 10. This indicates that the clinical remission rate of multiple FMTs is significantly higher than that of single FMT.

The safety of FMT in pediatrics CD is relatively good. The adverse events (AEs) mainly include nausea, vomiting and abdominal pain, and most of them are mild (Suskind et al., 2015; Goyal et al., 2018). Our recent study reports the short term safety of FMT in children, most AEs (88.5%) occurred within 2 days post-FMT, 91.4% of the AEs were self-limiting (Zou et al., 2022). This current study also shown that the short-term safety of FMT was very good. Although 7 out of 12 patients had FMT-related AEs, accompanied by two serious adverse events, most of the symptoms were mild. These symptoms may be related to poor intestinal mucosal barriers and the intestinal environment in children with CD.

The number of donor strains decreased within 1.5-3 months after FMT, and the theoretical effect of FMT decreased with a decrease in donor strains (Li et al., 2016). A recent study found that after three months of FMT treatment for IBS, the clinical response rate was 65% in the FMT group and 43% in the placebo group. However, the efficacy of FMT decreased significantly after 12 months and was similar to that of the placebo group (Johnsen et al., 2018). Therefore, we recommend periodic FMT to help maintain efficacy. To date, there have been several studies of repeated FMTs (Costello et al., 2019; Schierová et al., 2020; Wang et al., 2020), but few studies of periodic FMTs. Our study confirmed that periodic FMT within 10 weeks could significantly maintain long-term efficacy and help sustain mucosal healing in pediatric CD. In the FMT group of the study, at week 8- 10, eight patients received periodic FMTs in the FMT group, of which seven (87.5%) maintained clinical remission, six (75%) underwent endoscopic remission, and five (62.5%) had mucosal healing; meanwhile, three patients were treated with PEN (80.0%) only and without periodic FMT treatment, of which one (33.3%) was in clinical remission and 0 had mucosal healing. The results demonstrated that periodic FMT could be a safe, feasible, and effective treatment for children with active CD. To our knowledge, this is the first time that periodic FMTs has been used in the treatment of CD in children.

Our study has some limitations. This was not a randomized clinical trial that would have provided more important evidence. Additionally, we primarily focused on the clinical efficacy of repeated FMT, with relatively little attention paid to the variation in gut flora. Finally, the frequency of FMT required to maintain long-term clinical efficacy in pediatric CD requires a larger sample size.

In conclusion, this retrospective study demonstrated that repeated and multiple doses of FMT plus PEN had a beneficial effect in inducing and maintaining clinical remission and mucosal healing in newly diagnosed children with active CD. Therefore, FMT can be a potential first-line treatment regimen for active pediatric CD. Further prospective studies are required to verify these findings.

## Data availability statement

The original contributions presented in the study are included in the article/**Supplementary Material**. Further inquiries can be directed to the corresponding author.

## Ethics statement

The studies involving human participants were reviewed and approved by Medical Ethics Committee of Tongji Hospital, Tongji Medical College, Huazhong University of Science and Technology. Written informed consent to participate in this study was provided by the participants’ legal guardian/next of kin. Written informed consent was obtained from the individual(s), and minor(s)’ legal guardian/next of kin, for the publication of any potentially identifiable images or data included in this article.

## Author contributions

SS and ZH designed the research. BZ performed the literature search and data extraction and drafted of the manuscript, SL, XL and CD after FMT and follow-up. JH and MR collected the data. BZ provided scientific statistic analysis methods. SS and ZH conducted FMT. All authors contributed to the article and approved the submitted version.
